# Revumenib (SNDX-5613): a promising menin inhibitor for the management of relapsed and refractory acute myeloid leukaemia (AML)

**DOI:** 10.1097/MS9.0000000000001888

**Published:** 2024-03-18

**Authors:** Harmla Hussain, Syeda Mahrukh Fatima Zaidi, Syed Mohammad Hasan, Aelia Sarv Jahan, Burhanuddin Sohail Rangwala, Hussain Sohail Rangwala, Mirha Ali, Asma Ahmed Farah

**Affiliations:** a Department of Surgery Unit 6, Dr. Ruth K. M. Pfau, Civil Hospital Karachi; b Department of Medicine, Jinnah Sindh Medical University, Karachi, Pakistan; c Department of Medicine, East Africa University, Boosaaso, Somalia

Leukaemia is a generic term for various groups of blood cancers. It is defined as an abnormal production of leucocytes in bone marrow and lymphoid tissue. Broadly classifying, there are two main kinds of leukaemia: acute and chronic. Acute myeloid leukaemia (AML), the most prevalent and aggressive form of leukaemia among adults, has a poor prognosis and accounts for many deaths annually^1^. According to the cancer statistics for 2023, ~11 310 patients are likely to die of AML, and an estimated 20 380 new cases can emerge in the United States only, rendering the substantial prevalence of the disease^1^.

The current management of AML mainly comprises chemotherapy or allogeneic hematopoietic stem cell transplantation (HSCT). Cytarabine and Anthracycline are the drugs of choice in induction therapy of AML, in accordance with the standard of care^2^. The regimen includes seven days of continuous infusion of Cytarabine (100–200 mg/m^2^/d from 1st day–7th day) along with Daunorubicin (60 mg/m^2^/d from 1st day–3rd day)^2^. Higher doses of Cytarabine or Anthracyclines; or the addition of a third agent such as Gemtuzumab ozogamicin during induction therapy can be considered to attain optimal results. The administration of HiDAC consolidation therapy with Cytarabine (3 g/m^2^ twice daily on the 1st, 3rd, and 5th day) is recommended for younger patients who are not eligible for HSCT^3^. The treatment options for relapsed/refractory AML could be better outlined. However, several novel regimens and therapeutic agents are being investigated to induce long-term disease remission.

To complement the existing treatment of AML, a novel drug Revumenib, has been introduced, showing promising results in AML. SNDX-5613 (Revumenib) is an oral, potent, selective inhibitor of Menin-KMT2A interactions^4^. Menin is a nuclear protein that acts as a scaffolding platform for epigenetic regulators. It promotes leukemogenesis by increasing H3K4 trimethylation and HOX gene expression^5,6^. By inhibiting the Menin-KMT2A interactions, the drug suppresses the leukemogenesis in Nucleophosmin (NPM1) mutated AML, the most frequent genetic variation in adult AML, representing 30% of all cases of AML and Lysine methyltransferase 2A (KMT2A) rearrangements or mixed lineage leukaemia (MLL) occurring in up to 10% of AML^4,7^.

In a recent study published in *Nature,* Dr Issa announced the outcome of 1st in human phase 1/2 clinical trial, AUGMENT-101 of SNDX-5613 Revumenib (Menin inhibitor) in relapsed or refractory (R/R) AML patients^4^. A total of 60 adults and eight children were enroled in the trial. All the patients were massively pretreated with the median of four previous therapies, and 48% of the patients had undergone allogeneic stem cell transplants. Only 60 patients with KMT2Ar and NPM1 mutations were incorporated into the efficacy analysis. About 30% (18 out of 60) of these patients attained complete remission or complete remission with partial haematological recovery (CR/CRh), with 78% (14 out of 18) of them attaining minimal residual disease (MRD) negativity. The average period of response in patients who experienced CR/CRh was 9.1 months. The overall response rate (ORR) of Revumenib [CR/CRh/complete remission with incomplete haematologic recovery (Cri)/complete remission with incomplete platelet recovery (CRp)/morphologic leukaemia-free state (MLFS)] was 53%, with 12 patients undergoing stem cell transplant after successful remission.

The only notable side effects associated with Revumenib were prolonged QT interval, febrile neutropenia, nausea, and vomiting. Meanwhile, 11 out of 68 (16%) patients experienced grade 2 or less differentiation syndrome (DS), a life-threatening condition characterized by fever, arthralgia, leukocytosis, pleuropericardial effusion, and renal or respiratory failure in severe cases^8^. It is a common occurrence affiliated with anti-leukaemic therapies, especially in patients undergoing arsenic trioxide, all-trans-retinoic acid (ATRA), and isocitrate dehydrogenase inhibitors therapies^9–11^. All the cases were resolved following the administration of corticosteroids and hydroxyurea. None of the side effects were severe enough to discontinue the Revumenib therapy.

An alternate menin inhibitor, KO-539 Ziftomenib, is also being investigated in patients with relapsed or refractory AML. The result of the phase 1/2 clinical trial of Ziftomenib has shown an ORR of 40% and CRh of 35% in patients with NPM1 mutation AML, with a relatively poor outcome in patients with KMT2Ar AML, exhibiting an ORR of 16.7% and CRh of 11%. The drug has also been associated with an adverse effect of Differentiation syndrome of at least grade 3 severities, which was manageable with protocol guidance.

Nonetheless, several challenges and difficulties have arisen in the management of relapsed and refractory AML. The advancement of highly sensitive minimal or MRD assays has revealed a limitation in the current remission criteria, which were initially introduced in 1956^12^. This limitation is evident in the discrepancy between the perceived success of current induction therapy in achieving complete remission in the majority of patients and the harsh reality of median overall survival times of less than 2 years in individuals with AML^13^. Furthermore, resistance to traditional chemotherapy and targeted therapies, which can result in treatment failure and disease progression, is a major challenge^14^. Patients with relapsed or refractory AML face limited treatment options, particularly if they are not eligible for stem cell transplantation. However, even for those who are eligible, the effectiveness of HSCT in leukaemia is hampered by several factors, such as HLA disparities, graft-versus-host disease, and the risk of relapse^15^. Therefore, limited, targeted drug interventions are available for the treatment of AML with fewer adverse effects and better therapeutic benefits. This necessitates the need for larger powered clinical trials conducted on the different treatment options available.

Revumenib, therefore, in accordance with the established mechanism of action of Menin inhibition, has exhibited down-regulation of leukemogenesis with an increased expression of differentiation genes, exhibiting reversal of abnormal proliferation of cancer cells and reviving the optimal tissue function. Monotherapy with Revumenib has led to promising anti-leukaemic activity in mNPM1 and KMT2Ar AML, causing a sustained remission of disease and significantly contributing to the domain of investigational therapies in relapsed and refractory AML. Therefore, Revumenib has the potential to be a better substitution for the current medical treatment of refractory/relapsed AML. The drug is currently being tested in phase 2 of the AUGMENT-101 trial to achieve FDA approval to harvest its actual benefits in AML patients. The affordability and availability of the drug should be kept reasonable to facilitate the targeted population.

Even though Revumenib potentially seems like a better treatment option for refractory/relapsed AML, several possible challenges may also arise with the use of Revumenib. Revumenib, like many targeted therapies, carries the risk of developing resistance over time, which could reduce its efficacy in the long run^16^. For Revumenib to be approved by regulators and used in clinical settings, it is essential to carefully design regulated clinical trials with appropriate outcomes to precisely evaluate the medication’s safety and effectiveness in various subgroups of AML patients, both as monotherapy and in combination with other drugs. Understanding Revumenib’s toxicity profiles and possible side effects is crucial. Determining the ideal dosage, treatment duration, and administration schedule, especially for long-term use, is essential for successfully incorporating it into AML treatment regimens. This approach aims to maximize the therapeutic benefits of Revumenib while minimizing toxicity. In conclusion, Revumenib has the potential to be a useful addition to the arsenal of treatments for refractory/relapsed AML, potentially improving patient outcomes and quality of life by addressing these issues and following future research avenues.

## Ethical approval

Not applicable.

## Consent

Not applicable.

## Source of funding

Not applicable.

## Author contribution

The conceptualization was done by H.H. and B.S.R. The literature and drafting of the manuscript were conducted by H.H., S.M.F.Z., S.M.H., A.S.J., H.S.R., M.A. and A.A.F. The editing and supervision were performed by B.S.R. All authors have read and agreed to the final version of the manuscript.

## Conflicts of interest disclosure

The authors declare no potential conflicts of interest concerning the research, authorship, and/or publication of this article.

## Research registration unique identifying number (UIN)

Not applicable.

## Guarantor

Not applicable.

## Data availability statement

Not applicable.

## Provenance and peer review

Not invited.
